# The Erythrocytic Hypothesis of Brain Energy Crisis in Sporadic Alzheimer Disease: Possible Consequences and Supporting Evidence

**DOI:** 10.3390/jcm9010206

**Published:** 2020-01-12

**Authors:** Elena Kosenko, Lyudmila Tikhonova, Gubidat Alilova, Amparo Urios, Carmina Montoliu

**Affiliations:** 1 Institute of Theoretical and Experimental Biophysics of Russian Academy of Sciences, Pushchino 142290, Russia; ljudasik09@rambler.ru (L.T.); hells2012@yandex.ru (G.A.); 2 Hospital Clinico Research Foundation, INCLIVA Health Research Institute, 46010 Valencia, Spain; aurios@cipf.es (A.U.); carmina.montoliu@uv.es (C.M.); 3 Pathology Department, Faculty of Medicine, University of Valencia, 46010 Valencia, Spain

**Keywords:** Alzheimer’s disease, amyloid β peptides, brain energy crisis, erythrocytic hypothesis, red blood cells, restoration of energy metabolism

## Abstract

Alzheimer’s disease (AD) is a fatal form of dementia of unknown etiology. Although amyloid plaque accumulation in the brain has been the subject of intensive research in disease pathogenesis and anti-amyloid drug development; the continued failures of the clinical trials suggest that amyloids are not a key cause of AD and new approaches to AD investigation and treatment are needed. We propose a new hypothesis of AD development based on metabolic abnormalities in circulating red blood cells (RBCs) that slow down oxygen release from RBCs into brain tissue which in turn leads to hypoxia-induced brain energy crisis; loss of neurons; and progressive atrophy preceding cognitive dysfunction. This review summarizes current evidence for the erythrocytic hypothesis of AD development and provides new insights into the causes of neurodegeneration offering an innovative way to diagnose and treat this systemic disease.

## 1. Introduction

Alzheimer’s disease (AD) is a progressive systemic disorder that develops slowly attacking the brain, brain blood vessels, and peripheral tissues [1], as well as red blood cells (RBC) [2,3], platelets [4], and leukocytes [5], and is characterized by progressive deterioration in cognitive abilities including memory impairment, typically in the elderly.

In 95%–98% of cases AD develops sporadically, and according to statistics on this type of dementia, about 50% of people aged 80 have the disorder [6]. Unless a way is found to halt progression of this disease, and if the population continues to grow, it is believed that by 2050 the number of people worldwide with this illness will be close to 115 million people, 7.7 million new patients per year, meaning that someone in the world will develop AD every 4 s [7].

Although during recent decades significant progress has been made in presenting different hypotheses of sporadic AD [8,9,10,11,12,13,14,15,16,17,18,19], the underlying mechanisms of the disease need further scrutinity.

The “amyloid cascade hypothesis” [20] has greatly influenced research over the last twenty years with considerable effort devoted to designing new anti-amyloid drugs, and although there is still no cure for AD [21,22], this hypothesis remains the most prevalent. Nevertheless, the key points of the hypothesis suggesting a central role for amyloid plaques in disease development and considering the brain and its age-related changes independently of the blood flow are paradoxical rather than convincing. The main reason is that the idea behind the “amyloid cascade hypothesis” is not consistent with the known axiom stating that even a short pause in glucose and oxygen inflow to the brain causes its damage and chronic deficiency in these substrates can ultimately lead to irreversible brain damage affecting cell viability and activity, and further promoting permanent cognitive impairment [23,24]. Understanding the mechanisms through which aerobic glucose metabolism is disturbed in the brain is clearly of paramount importance.

Many mechanisms are involved in impaired glucose oxidation in the brain affected by AD [25]. It is noteworthy, however, that earlier studies were mostly conducted using autopsy brains from AD patients as the object of examination [26,27], making it difficult to unravel the true mechanisms of the failure in brain energy metabolism, since many factors influence results obtained using postmortem brain tissues, including the agonal stage, brain storage time and temperature protocol, and lactate accumulation [28,29]. Therefore, no possible correlation between the ascertained causes of the impaired glucose utilization and clinical symptoms of the disease has been identified [26]. By contrast, with functional brain imaging techniques for measuring brain metabolic fluxes in vivo [30,31] it is possible not only to identify the brain regions with significant reductions in glucose metabolism but also to predict memory impairment in people with normal cognitive functions long before the clinical symptoms appear [32,33,34,35,36]. Thus, there is evidence supporting the view that the reduction in blood-carrying energy substrates, particularly glucose and oxygen observed in vivo in AD, results directly from cerebrovascular pathology [37]. This ultimately leads to energy collapse followed by the functional brain failure, and as a consequence, cognitive impairment [38,39].

Another essential factor contributing to brain oxygenation is the oxygen-carrying capacity of red blood cells. It is known that the role of RBCs in tissue oxygen delivery depends on their intracellular metabolism, primarily via energy metabolism and antioxidant status [40,41], which regulate hemoglobin oxygen affinity [42] and NO-dependent hypoxic vasodilation [43,44,45].

Numerous studies have shown that normal RBC parameters might be significantly affected in AD patients. For instance, in AD patients RBCs have been found to exhibit morphological changes [46,47], leading to decreased deformability [48], perturbations in the physical state of membrane proteins [49,50], and oxidant/antioxidant equilibrium [51,52,53,54]. These changes are in some way due to the destabilization of intracellular metabolism in erythrocytes [55,56,57,58]. However, the mechanisms underlying the foregoing alterations in RBC metabolism in AD patients are not fully understood. Furthermore, AD is still thought to be exclusively a brain disorder, so a link between disruptions of the endogenous metabolic pathways in circulating erythrocytes that slow down oxygen release, and brain energy crisis manifested as poor brain aerobic glucose oxidation which may precede neurodegeneration is not sufficiently discussed in the literature.

In this article, we review current evidence in support of the widely known amyloid cascade hypothesis and seek to uncover new perspectives on the cause of sporadic AD development, considering brain amyloids outside pathology and suggesting a new mechanism by which neurodegeneration may occur.

Based on our and available published data [46,47,48,49,50,51,52,53,54] we hypothesize that abnormalities in endogenous glycolytic, antioxidant, and transport pathways in circulating erythrocytes in individuals entering old age [59] disrupt red blood cell function associated with oxygen supply to the brain. This causes deterioration in the aerobic oxidation of glucose leading to the nerve cell degeneration and impaired cognitive processes observed in AD [25].

This assumption is not in contrast with the vascular hypothesis predicting neurodegeneration as a result of impaired blood circulation and brain hypoperfusion [60,61,62]. On the other hand, it clearly indicates the possible existence of another unknown mechanism which restricts oxygen supply to the brain, and therefore participates in the development of hypoxia and neurodegenerative processes specific to AD.

Consequently, we believe that changes in endogenous biochemical parameters in erythrocytes can be considered both an indicator of cell state in the central nervous system, and a new target to develop innovative treatment methods for systemic disease such as AD.

This suggestion provides a basis for developing individual innovative technologies to restore energy metabolism, the antioxidant system and other impaired endogenous biochemical pathways in erythrocytes [63]. This will help improve RBC function to provide an adequate oxygen supply to the brain and normalize aerobic glucose utilization.

## 2. Evidence that Contradicts the Fundamental Principles of the Amyloid Cascade Hypothesis

### 2.1. Brain Amyloid Plaques and Soluble Aβ Oligomers are Unspecific for AD

The fact that cerebral amyloidosis is generated during many human diseases has been knоwn for more than a century, when amyloids (Aβ) were found in the brain and cerebral vessels of a yоung woman with cognitive dysfunction caused by traumatic injury to the head [64]. Almost simultaneously, researchers discovered that Aβ deposits are formed and stored in the brain in patients with other pathologies [65,66].

These facts indicate that cerebral amyloidosis does not depend on age and may occur in people without dementia. The latest research results are consistent with data obtained in previous studies which indicate that Aβ peptides are non-specific for AD and accumulate in the brain of children and adults with many acute and chronic central nervous system disorders [67,68,69,70,71,72,73,74,75], as well as in normal non-demented elderly persons [76,77]. Further, in some individuals, Aβ levels in the brain are comparable to or even higher than is typical in AD patients [78,79]. Moreover, a close correlation between severity of cognitive dysfunction and total amyloid volume in the brain of AD patients is not generally detectable [80,81,82,83,84]. Taken together, these results suggest that neither the density of amyloid plaques nor the absolute amount of amyloids can predict cognitive dysfunction impairment or disease onset [85]. These findings contradict the amyloid cascade hypothesis, which states that “deposition of amyloid β protein, the main component of the plaques, is the causative agent of Alzheimer’s pathology and that the formation of neurofibrillary tangles, cell loss, vascular damage, and dementia follow as a direct result of this deposition” [20].

Soluble Aβ oligomers, which according to the modified amyloid cascade hypothesis are the initiators of the fatal cascade in AD [86,87], have also been detected in the brains of healthy elderly individuals [88,89]. Moreover, the increase in soluble Aβ level in AD patients compared with healthy normal subjects is negligible [90]. At the same time there is evidence indicating that the accumulation of soluble Aβ oligomers in the brains of patients with AD is episodic with no direct correlation between this accumulation and the appearance of clinical symptoms of the disease [91]. In addition, after the discovery of soluble Aβ oligomers in brains of patients with Down’s syndrome [92] and traumatic brain injury [93,94] it can be assumed that soluble Aβs like aggregated Aβ peptides are unspecific fоr AD prompting a search for new pathogenic factors.

### 2.2. Aβ Neurotoxicity: Which Peptide Is a Therapeutic Target

The toxicity of amyloid peptides is the main argument supporting the amyloid cascade hypothesis. However, it must be acknowledged that data on toxicity of amyloid peptides are obtained exclusively from in vitro experiments [95,96,97], while participation of amyloids in neuronal death has not yet been demonstrated in vivo.

Indeed, numerous studies conducted in vitro have shown that amyloids induce changes in neurons leading to numerous disorders incompatible with cell vitality. As an example, in primary neuronal cultures neurotoxicity is associated with channel formation in the membrane [98,99], increased metal-dependent production of reactive oxygen species, and oxidative stress [100]. Additionally, interaction between amyloids and cells leads to changes in activity of membrane-bound proteins [101] and receptors [102], in dynamic properties of lipids [103], and loss of phospholipid asymmetry and integrity [104], that in turn increases membrane fluidity [105] and permeability [106]. The damaging effect on the membrane structures causes dysfunction of the ion pumps and a consequent increase in intracellular calcium [107] triggering a glutamate-dependent pathological cascade that results in further cell death [108]. Interestingly, in vitro both soluble Aβ oligomers and aggregated Aβ forms induce cell death in virtually any cultured cell type, and cell death occurs within a few minutes of exposure to amyloids. A number of questions arise about the significance of these data. For example, if amyloids are so toxic that even a short-term interaction between the cell and amyloid causes the death of the former, how can these amyloids be “silent” in the brain of AD patients for so long time (15–25 years) before the appearance of the first clinical symptoms of the disease [109,110]? If amyloids are toxic, how can people with this disease live to a very old age and make up the largest group of total cases people aged over 95 [111]? And more, if amyloids located in the brain of elderly individuals with normal memory are toxic why do these toxic substances not affect intellectual capacity, work ability and cognitive processes to a ripe old age? It is clear that the in vitro study results do not always provide easy answer to any of the questions raised.

The issue of amyloid toxicity was not clarified by findings from studies carried out in vivo either. It has been shown that isolated plaque cores from postmortem AD brains [112] and synthetic Aβ fragments produced AD-like neuropathology in vivo [113,114]. A similar effect was observed for soluble Aβs [115], which according to the reformulated “oligomeric amyloid hypothesis” are the principal neurotoxins [116]. In addition, both peptides disrupted learned behavior in normal animals [117,118]. These observations again raised the question of which hypotheses is correct and what peptide should be a therapeutic target, to which the answer would appear to be an aggregated one as all transgenic mouse models [119] as well as transgenic *Drosophila* models [120], or even transgenic strain of the nematode *Caenorhabditis elegans* [121], used for screening of anti-amyloid drugs, develop only Aβ aggregates [122], ultimately leading to plaque formation [123].

The amyloid cascade hypothesis [20] and the oligomeric amyloid hypothesis [116] evidently contradict each other [124], thereby lowering the level of a huge body of data obtained via in vitro and in vivo studies. Thus, the question remains open as to the toxicity of amyloids in vivo and the expediency of designing new drugs based on contradictory hypotheses which are associated with a pathogenic factor whose toxicity has not yet been proven [22].

### 2.3. Can Transgenic Rodents Be Used as a Suitable Model for Sporadic AD

Returning to the amylоid cascade hypоthesis, which was originally postulated for the genetic form of the disease [21], it should be kept in mind that detection of APP and presenilin gene mutations [125] in the genetic form of AD was the only strong evidence underlying the amyloid hypothesis [116]. However, there was no evidence proving that amyloids are the only cause of this disease, especially its sporadic form [123], in which there are no such gene mutations, but instead increased amyloid synthesis which leads to accumulation, aggregation and amyloid plaque formation, characteristic of genetic disease. Interestingly, that by creating an amyloid cascade hypothesis, D. Hardy assumed that “The mutations in APP so far described are responsible only for a small proportion of cases of Alzheimer’s disease. Indeed, most cases of Alzheimer’s seem to occur in a sporadic fashion, suggesting that there must be other causes of the disease. The cascade hypothesis suggests that other causes of Alzheimer’s act by initially triggering APP deposition. This deposition could be caused by an induction of the APP gene through an interleukin-mediated stress response because APP increases in response to a number of neuronal stresses” [20]. A review of the literature sheds no light on when or by whom the role of genetic mutations was first considered in the pathоgenesis of sporadic form of AD. Apparently confusion has arisen concerning the hypothesis: it was proposed only for the genetic form of AD, and all anti-amyloid preparations were tested on transgenic mice, using the so-called genetic mouse models of AD, but clinical trials only recruited patients with sporadic disease [21], without mutations in the abovementioned genes. Further, despite the fact that all types of transgenic models perfectly imitate the amyloid aspect of AD [126], the relationship postulated by the amyloid cascade hypothesis between enhanced amyloid formation and accumulation of hyperphosphorylated tau protein, memory impairment, and neuronal death, is not generally confirmed, using transgenic models [127,128,129]. Moreover, the memory impairment and neuron death that occur before the appearance of amyloids in the brains of transgenic animals [130,131] may indicate that artificially introducing alien genetic information into the genome of animals might have unexpected pathological effects on the brain and the body as a whole. This prediction is verified by the hippocampal (and other brain regions) hypometabolism and atrophy observed prior to plaque formation [132] and premature deaths in transgenic mice [133], most probably due to the cerebral energy crisis resulting from artificial introduction of alien transgene rather than from accumulated amyloid peptides [134]. Moreover, this fact points to the presence of unrevealed problems that usually arise when creating transgenic models. In fact, overexpression of APP in transgenic animals can dramatically disrupt the mitochondrial function [135] resulting in enhanced oxidative stress [136], uncontrolled release of neurotransmitters [137] and cell death via the apoptotic pathway. This indicates a need to identify the pathological changes occurring in the brains of transgenic animals prior to augmented amyloid formation and whether changes in the behavioral responses of transgenic animals result from the accumulation of amyloids, rather than APP-inducing brain hypometabolism. The genesis of degenerative processes and memory impairment before the appearance of amyloids in the brain, and the premature death of transgenic animals preclude using these animals as adequate models of sporadic AD [126]. This view is supported by a large body of clinical trials showing negative effects, and even death in sporadic AD patients who received anti-amyloid preparations [22,138,139,140], despite their therapeutic effect having been confirmed in transgenic mice [141,142,143]. It is clear that any animal model has its advantages and disadvantages, but patients’ lives depend on a model that meets the main requirement of equivalent action in animal models and patients and development of safety drugs. 

Certainly, the approach in research to the problems of the sporadic form of AD seems poorly planned demonstrating a logical gap in the “hypothesis–objective–test” chain which leads to a focus only on amyloids. In fact, it is clear that if Aβ deposition occurs in response to cerebral hypometabolism in even young transgenic animals [132], and in elderly AD individuals [144,145], a search for factors that trigger pathological processes in murine and human brains, which in turn lead to APP overexpression and Aβ accumulation, is of crucial importance in current biomedical research. 

## 3. Brain Energy Crisis and Aβ Accumulation: Cause or Consequence

Glucose is the main brain energy metabolite, the precursor of substrates that are oxidized in the mitochondrial tricarboxylic acid cycle to form ATP [146]. Glycogen is the only endogenous source of glucose in the brain, but since its reserves are extremely small and the brain is unable to synthesize glucose de novo quickly enough [147], brain cell viability and its myriad functions are completely dependent on a continuous supply of glucose via blood flow. 

In addition to ATP, other important energy substrates, for example, lactate produced by glucose metabolism are used by neurons as an additional energy substrate when metabolic activity is high [148,149]. Glucose carbon can be included in lipids [150], proteins [151], and glycogen [152] and is also a precursor of some neurotransmitters such as γ-aminobutyric acid, GABA [153], glutamate [154], and acetylcholine [155] as well as nicotinamide adenine dinucleotide phosphate reduced (NADPH) essential for maintaining brain antioxidant capacity [156].

Thus, glucose entering the brain from the blood is the main substrate known to regulate numerous brain functions, including brain activity, learning, memory [157], and nerve cell viability in general [158,159].

Glucose oxidation in the brain is suppressed with age [160,161], but in individuals without chronic diseases or silent brain pathology, a decrease in metabolic rate usually comes with almost no symptoms, and elderly people can live long enough in relatively good physical and mental health. By contrast, in sporadic AD aerobic oxidation of glucose and the rate of ATP formation in the brain decrease dramatically [162] resulting in damage to nerve cells and influencing neuronal function and mental states [163,164]. 

As is known, neurons in different brain structures are not equally sensitive to glucose shortage. Therefore, it is not a surprise that in AD all glucose-dependent neurons, localized in the hippocampus and neocortex [165], involved in the process of long-term memory storage [166], are damaged to a large extent in a state of glucose deprivation. Interestingly, significant accumulation of amyloid peptides is revealed in these brain regions in AD patients [167] suggesting that glucose deficiency is related to increased amyloid formation in the brain. 

It is obvious that the causes of amyloid proliferation in the brain in the sporadic form of AD should differ from those described for the genetic form of the disease, if only because APP and presenilin gene mutations are not observed in sporadic AD. 

Assuming there is a relationship between disturbances in glucose metabolism in the brain and amyloid formation, the question then arises as to which process occurs first: the formation of amyloids, which have toxic effects on neurons and disturb all types of metabolism, including energy thus resulting in neurodegeneration; or formation of amyloids in response to chronic glucose hypometabolism which serve as a beacon, signaling disturbances in energy metabolism that trigger neuronal and cognitive dysfunction [168]. However, based on the assumption that the disruption of glucose utilization in the brain is a primary cause of neurodegeneration, it is expected that APP up-regulation and accelerated Aβ formation and accumulation should occur irrespective of age or cause of glucose deficiency. Taking the predominant amyloid cascade hypothesis into account, this assumption is at least a little controversial, but it is confirmed by a huge body of evidence showing that APP overexpression, leading to intensive Aβ production, is observed in multiple pathologies associated with the breakdown of energy metabolism in the brains both in humans and animals. Thus, enhanced amyloid formation and accumulation occurs in the brains of people of different ages during: hypoxia [169], human and animal head trauma [170,171], spinal cоrd injury [172,173], neurоinflammatiоn [174,175], middle cerebral artery оcclusion [176], general anesthesia [177], influence of bacterial agents [178,179] and other examples [25]. In fact, analysis of the above pathologies leads directly to the conclusion that there is steady damage of brain cells resulting from a number of different causes. For example, anesthesia can significantly disturb hemodynamics, reducing the brain’s oxygen and glucose absorption rate [180] thereby leading to severe hypoxia, a sharp inhibition of aerobic oxidation and neuronal activity [181]. Interestingly, increased APP processing and amylоid formation occur in the brain a few hours after administering isoflurane as an anesthetic [182]. Attenuated intake of glucose and oxygen to the brain together with the oxidative stress observed in ischemia-reperfusion also initiates APP processing and amyloid formation in damaged brain structures [183]. Hypoglycemia-limited brain glucose intake and also promote amyloid accumulation [184]. Taken together, these data confirm that Aβ peptides could accumulate in the brain any time when brain cells are destroyed due to a paucity of energy resources [144,185]. Some scientists therefore believe that the initial Aβ formation can be considered an adaptive reaction [186,187], protecting neurons against further damage [188]. If this hypothesis is true, we must accept that the formation of amyloid plaques in the AD brain is not the start of a suicide program, but rather a signal indicating a disruption of brain metabolism and maybe even not the beginning, but the continuation of pathological process that may have gone unnoticed for several years at least [189]. Thus, unveiling the mechanisms of glucose metabolism disruption that underlie APP up-regulation in the sporadic form of AD is a task of current concern in medicine and biology as a precursor to identifying risk factors for disease development and detecting new, alternative targets (other than amyloids) for therapeutic effect.

## 4. The Possible Role of Erythrocytes in Neuronal Aerobic Glucose Metabolism Crisis in AD: The Erythrocytic Hypothesis

Erythrocytes are the only cells able to carry oxygen and maintain aerobic utilization of glucose in tissue. In fact, many people are already well informed about red blood cells in a simplified way, viewing them only as small bags filled with hemoglobin that binds oxygen reversibly. Notably, a remarkable series of studies has previously demonstrated a pivotal relationship between RBC metabolism and binding, transport, and delivery of oxygen to tissues. Curiously, the first reports about this relationship were published more than 40 years ago [40] suggesting that these scientific findings are largely ignored. As a consequence, there is limited information regarding the interactions between metabolism and erythrocyte function. Traditionally, red blood cell indices, hemoglobin quantities, hematocrit test, sedimentation rate, average volume, and morphological changes etcetera are used to diagnose different diseases, but it is unknown how changes outside the normal range in the above-measured parameters affect RBCs’ ability to deliver oxygen to tissues. It follows then that there is no tool for diagnosing metabolic illness in RBCs themselves, a disorder that may appear influenced by pathological factors present in circulation [190].

Passing through the lungs, RBCs are exposed to oxidative stress, in the kidneys which are characterized by hypertonic condition, the cells are the target of osmotic shock. To get through tight capillaries, erythrocytes experience significant mechanical compression, having to pass through capillaries with diameters smaller than their own [191]. It has been proven that cells with impaired intracellular energy metabolism are unable to withstand such exogenous attacks, becoming damaged and lysed directly into the bloodstream as evidenced by the permanent presence of cell-free plasma hemoglobin [192].

Another consequence of erythrocyte “disorder” is an increase in oxygen affinity to hemoglobin, leading to the tissue hypoxia [193] and, in particular, cerebral hypoxia. In order tо fulfil their function, RBCs should be healthy which depends primarily on their metabolic and antioxidant status contributing to adequate oxygen binding capacity. For this, all metabolic pathways in mature erythrocytes should be strictly regulated and proceed at the rate necessary to maintain their viability and functional activity. One of these processes is anaerobic glycolysis, the only source of ATP production. Pentose phosphate shunt is necessary for NADPH formation, used in combined enzymatic reactions that catalyze glutathione reductase and glutathione peroxidase. These enzymes are antioxidant and responsible for maintaining low levels of hydrogen peroxide and therefore preventing methemoglobin formation. Erythrocytes also need sufficiently high activity of other antioxidant enzymes such as superoxide dismutase, which maintains a safe level of superoxide radical (O_2_^•−^), glutathione transferase, which is involved in xenobiotic detoxification [194], catalase and peroxiredoxin-2-peroxidase, which destroy hydrogen peroxide. Red blood cells are not able to synthesize ATP de novo, and energy homeostasis is maintained during high energy demand due to salvage pathways by which already existing nucleosides and purine bases can be recycled to produce adenine nucleotide triphosphates [195].

For the production of 2,3-diphosphoglycerate (2,3-DPG), the main metabolite that facilitates the release of oxygen from hemoglobin to tissue [196], and to maintain its steady state concentration, erythrocytes possess a unique glycolytic bypass called a Rapoport–Luebering shunt [42]. The activity of this pathway depends on the rate of glycolysis and a relative deficiency of enzymes catalyzing the initial reactions of glycolysis may cause insufficient synthesis of 2,3-DPG and affect the ability of RBC to release oxygen, leading to tissue hypoxia [197]. Therefore, intact intracellular metabolic pathways of erythrocytes are undoubtedly a major factor responsible for adequate oxygen delivery and release to tissues.

We have recently demonstrated differences in the parameters of various metabolic pathways in erythrocytes of patients with Alzheimer’s disease (AD group) and patients with non-Alzheimer’s dementia (NA group). We found that in erythrocytes of elderly control subjects (AMC, age-matched controls), glycolysis and cation transport were sharply inhibited, as evidenced by a significant decrease in all glycolytic enzyme activities, a decrease in the rate of glucose consumption, ATP concentration, the rate of pyruvate and lactate formation, and a sharp decrease in Na^+^, K^+^-ATP-ase activity compared to young adult controls (YAC). In the erythrocytes of AD and NA patients, these parameters changed to the same extent and significantly exceeded the parameters not only of AMC, but also of the YAC group indicating an increase in ion fluxes leading to acceleration of RBC glycolytic pathway [198,199]. Given that the active transport of cations through the erythrocyte membrane is controlled by the rate of ATP formation during glycolysis, up-regulation of Na^+^, K^+^-ATP-ase accompanied by a decrease in the concentration of ATP in RBCs of AD patients can be attributed to an imbalance between ATP formation and ion pumping, caused by increases in Na^+^ input in erythrocytes of AD patients [200]. Despite glycolysis activation, this imbalance ultimately promotes an increase in adenylate catabolism as confirmed by ADP and AMP accumulation, a decrease in the cellular energy charge and the total content of adenine nucleotides. The enzyme activity of the Rapoport–Luebering shunt, in particular, diphosphoglycerate phosphatase (DPGP-ase), that regulated the steady state levels of 2,3-DPG, increased simultaneously with a significant decrease in 2,3-DPG concentration in erythrocytes of the AD group compared to both age-matched controls (AMC) and young adult controls (YC). Clearly, the affinity of hemoglobin for oxygen is influenced not only by 2,3-DPG, but most likely by other factors such as pH level, PCO_2_, PO_2_, Cl^−^, conformations of hemoglobin and temperature. However, the correlation between the concentration of 2,3-DPG in erythrocytes and the degree of hypoxia in tissues, and particularly in the brain, has been revealed in many pathological states of human beings and animals [201,202,203]. Taken together, the results obtained suggest that chronic intensification in the rate of cationic traffic and plasma membrane permeability observed in the RBCs of AD patients, could trigger a cascade of pathological reactions in erythrocytes. This then leads to accelerated hydrolysis of ATP and 2,3-DPG, responsible for regulating the affinity of hemoglobin for oxygen, one possible explanation for inadequate oxygen supply to the brain, glucose hypometabolism, and cognitive abnormalities in AD. We have also found that changes in many metabolic pathways are already present in the erythrocytes of healthy individuals entering old age but without memory problems. Thus, the cells of the AMC group were marked by disturbed homeostasis of adenine nucleotides, a slight but significant decrease in 2,3-DPG concentration [59]. Similarly, there were no differences in pro-oxidant levels [199] between AMC, AD, and NA groups, while antioxidant enzyme activity, considerably reduced in RBCs of the AMC subjects, further decreased with dementia [198].

Our findings are largely consistent with data from other researchers who show the same direction of disturbances in erythrocytes of normal elderly people and patients with AD [204]. They also indicate that the disturbed metabolism and reduced antioxidant capacity in RBCs are not hallmarks of the terminal illness, but are indicative of stable and permanent changes in intracellular metabolism in erythrocytes of the elderly, which under the influence of unknown factors may lead to the pathological lesions encountered in AD patients. One factor very likely to contribute to altered intracellular metabolic pathways and RBC morphology in older people is enhanced entry of Na^+^ into the cells [205].

It is clear, therefore, that increased activity of Na^+^, K^+^-ATP-ase in the erythrocytes of AD patients can be considered an adaptive reaction to the increased entry of this cation into cells. At the same time, in chronic conditions, constant and multiple activation of the enzyme cannot completely compensate for the increased plasma membrane permeability that leads to pathological consequences which negatively influence RBCs’ energy metabolism and antioxidant defence system, enhancing the hydrolysis of ATP, 2,3-DPG, oxidative stress and proteolysis. The combined effect of this damage disrupts the function of erythrocytes associated with oxygen delivery, and also causes changes in the morphological property of erythrocytes, decreasing their ability to deform and thus lowering the threshold for development of neuropathology.

Our erythrocytic hypothesis is thus based on the obvious link between the state of intracellular metabolism of erythrocytes and its functional ability. We suggest that abnormalities in glycolytic, antioxidant and transport pathways can disrupt the erythrocyte function associated with oxygen delivery to the brain and cause disruption of the aerobic oxidation of glucose, leading to degeneration of the nerve cells and impaired cognitive function as observed in AD.

It is obviously necessary to elucidate what factors (endogenous and exogenous) cause the premature decrease in the reserve potential of erythrocytes to withstand stress that they constantly undergo, while circulating from the lungs to the tissues.

## 5. Encapsulation of Missing Enzymes in Erythrocytes: A Way to Reconstruct Energy Metabolism

Erythrocytes are known as cells that can be loaded with different kinds of substances (drugs, enzymes, metabolites, cofactors, etc.) through methods based on the hypotonic hemolysis-resealing procedure that allows to design intact and fully viable erythrocytes [206,207]. Based on the above methodоlogy we developed a procedure for encapsulating missing enzymes together with the substrates and cofactors involved in the energy metabolism and antioxidant defense system. Our findings show that after reinjection into rat blood, enzyme-loaded erythrocytes were able to retain their integrity, normal energy metabolism, and survive in blood circulation for a long time [25].

This biotechnological approach provides an extraordinary opportunity to develop innovative medications for individual use that will help specifically to treat the endogenous metabolic abnormalities in the red blood cells and restore adequate oxygen supply to the brain.

## 6. Conclusions

More than 100 years have passed since Alzheimer’s disease was identified, yet the pathogenesis of the disease remains unknown. Taking into account the role of the vascular hypothesis in AD pathogenesis, we assume that among the additional causes of pathological brain hypoperfusion typical in AD are structural and biochemical changes in erythrocytes, leading to inadequate oxygen supply to the brain, which inevitably precedes brain atrophy, developmental disabilities and dementia. Therefore, change in biochemical parameters in erythrocytes can be considered both an indicator of the cell state in the central nervous system, and a new target to develop innovative methods to treat this systemic disease.

The question of whether cerebral vascular damage or RBC damage is the most important cause of cerebral hypoxia is beyond the scope of this study. It is needless to say that both pathologies are intrinsically closely related to each other. However, since erythrocytes serve as the only oxygen carriers and their ability to bind, transport and deliver oxygen to tissues depends, primarily, on energy metabolism and antioxidant systems, we believe that disturbance of the endogenous processes in these cells most likely has a dramatic destabilizing impact on aerobic glucose metabolism in the brain and promotes dementia development. This assumption does not contradict the known pathological consequences of vascular damage, that leads to impaired blood circulation and brain hypoperfusion. At the same time, it clearly indicates the possible existence of additional unspecified mechanisms, restricting oxygen supply to the brain and therefore participating in the development of hypoxia and neurodegenerative processes specific to AD.

We therefore suggest that a combination of revascularization and restoration of metabolism in erythrocytes which maintain glucose/oxygen-dependent normal neuronal function could prevent brain cells from the destructive effects of energy limitation and hypoxia, and avoid dementia development. A thorough and detailed study of erythrocyte metabolism and morphology is needed not only to identify potential risk factors, but also to shed light on the molecular mechanisms that limit oxygen supply to the brain. This knowledge is important to prevent the development of hypoxia-induced neurodegenerative processes typical not only of AD, but also of many other diseases with different etiologies, and characterized by varying degrees of cognitive dysfunctions. 
